# Ovarian Cancer Was Discovered in Sister Mary Joseph's Nodule

**DOI:** 10.1155/2022/5131705

**Published:** 2022-06-29

**Authors:** Mouna Kouira, Imen Bannour, Mohammed Raouf Ben Abdesslem, Nihed Abdessayed, Badra Bannour

**Affiliations:** ^1^Department of Gynecology and Obstetrics, University Hospital Farhat Hached, Faculty of Medicine Ibn Al Jazzar, University of Sousse, Sousse, Tunisia; ^2^Pathology Department, University Hospital Farhat Hached, Faculty of Medicine Ibn Al Jazzar, University of Sousse, Sousse, Tunisia

## Abstract

**Introduction:**

Sister Mary Joseph's nodule (SMJN) is a rare illness characterized by an umbilical mass caused by tumor metastases in the abdomen or pelvis. The most common main site of SMJN in women is ovarian cancer. *Case Presentation*. A 73-year-old woman with no pathological history came to our emergency room with a one-month history of umbilicus enlargement. A 9-centimeter uncomfortable umbilical swelling with hard consistency was discovered during a clinical examination. An ovarian tumor with several local expansions was seen on an abdominal CT scan. It was linked to peritoneal metastases, one of which extends via a supraumbilical hernial orifice and into intestinal tissues in the same hernia sac. The umbilical tumor was removed from the patient. A moderately differentiated serous carcinoma with ovarian origin was identified in a periumbilical site on histological testing.

**Conclusion:**

The presence of an SMJN is a rare but significant issue that clinicians must examine because it is associated with a bad prognosis. Early detection and diagnosis of the original lesion can lead to more effective treatment and a higher rate of survival.

## 1. Introduction

Sister Mary Joseph's nodule (SMJN) is a rare disorder characterized by an umbilical mass caused by tumor metastases in the abdomen and/or pelvis [1]. According to epidemiological research, this condition is more common in women, accounting for 1 to 3% of all intra-abdominal and pelvic malignancies [2].

The most common main site of SMJN in women is ovarian cancer [3]. SMJN might take the form of a soft, harmless nodule or a firm, painful mass. Its size ranges from 1 to 10 cm [4]. The presence of the SMJN is linked to a poor prognosis, with overall survival ranging from 2 to 11 months from the time of diagnosis and a 5-year survival rate ranging from 5 to 15% [5].

The purpose of this case is to discuss a case of Sister Mary Joseph's nodule as a misdiagnosed ovarian cancer metastasis.

## 2. Presentation of the Case

A 73-year-old woman appeared with a firm umbilical mass that had been increasing for a month and had no history of an umbilical hernia. The patient was initially unconcerned about the lump because it was painless. The umbilical tumor had become uncomfortable two days before the presentation, causing the patient to seek medical help. She does not have any medical history.

**Figure 1:**
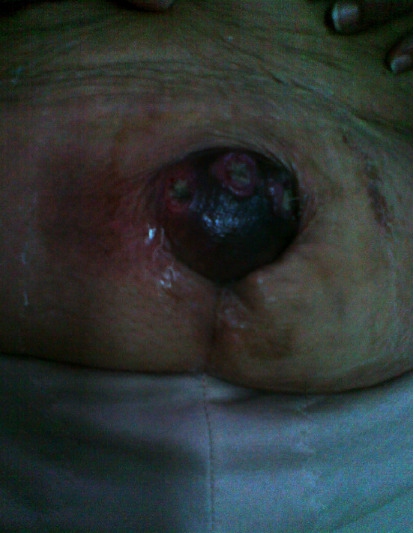
A 9-centimeter inflammatory and painful umbilical tumor with a hard consistency.

A healthy-looking woman was discovered during the physical test. An inflammatory and uncomfortable umbilical mass of 9 cm with hard firmness was discovered during the abdominal exam (Figure 1). It also revealed a 10-centimeter left inguinal adenopathy that was painless, noninflammatory, and firm to the touch. The rest of the medical examination went smoothly.

A 12 cm suspicious tumor in the left ovary was discovered by pelvic transvaginal ultrasonography.

We opted to finish with a pelvic scanner because of the importance of the pelvic mass, which exceeds the limits of ultrasound.

**Figure 2:**
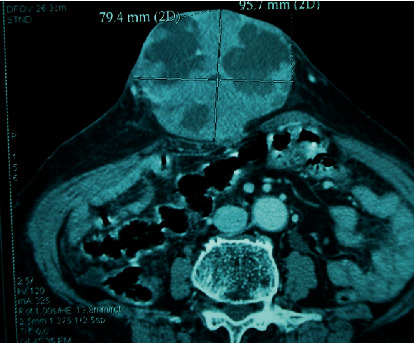
An ovarian tumor with intraperitoneal metastases, one of which extends through a hernial supraumbilical orifice, is shown on a CT scan of the abdominal cavity and pelvis.

The patient received an abdominal-pelvic computed tomography (CT) scan, which revealed a 12 × 11 cm left ovarian tumor with several local expansions. It was linked to intraperitoneal metastases, one of which is 9 × 8 cm in size and extends through a supraumbilical orifice with digestive processes in the same hernial sac (Figure 2).

The results of the biochemical tests revealed a normal total blood count and electrolytes as well as a high CA-125 level (200 UI/ml).

The patient was admitted to the hospital, and the diagnostic and treatment options were addressed with the patient and a multidisciplinary team. We first performed a resection of the umbilical mass with omentum reintroduction into the abdominal cavity and the removal of any remnant malignancies.

**Figure 3:**
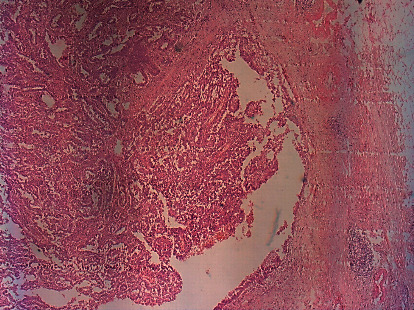
The umbilical nodule's microscopic features reveal a carcinomatous growth of glands and papillae lined by cuboidal cells with severe nuclear atypia (HEX 100).

The resection margins were devoid of tumor, and histological evaluation revealed a periumbilical site of a moderately differentiated serous carcinoma compatible with ovarian origin (Figure 3).

We made the decision to perform an exploratory laparotomy. During surgery, a solid mass measuring 12 cm was discovered on the left ovary, along with a tiny amount of ascites. There were also intra-abdominal and omentum metastatic lesions found. Total hysterectomy, bilateral salpingo-oophorectomy, bilateral pelvic lymphadenectomy, and omentectomy were the procedures performed.

Histopathology revealed an ovarian serous carcinoma, which was also found in the umbilical tumor. The lymph nodes in the inguinal and iliac arteries were positive, and the omentum had metastatic disease. As a result, the definitive diagnosis was ovarian cancer stage IV B (FIGO ovarian cancer staging).

The recovery went smoothly, and the patient was discharged a week following surgery. Chemotherapy with carboplatin (starting dosage AUC 5–6) and paclitaxel (175 mg/m^2^) was started one month after hospital discharge and continued every three weeks for six cycles.

Currently, the patient has completed 12 months of treatment and gets clinical examinations and tumor markers every three months. There was no clinical recurrence, and CA-125 levels gradually declined and returned to normal after two years.

## 3. Clinical Discussion

SMJN was named after Sister Mary Joseph Dempsey, who worked as a surgical assistant for Dr. William James Mayo. She was the first to link a metastatic umbilical nodule to abdominopelvic cancer [6]. It was first mentioned in print in 1864 [1].

SMJN is a rare condition caused by a primary neoplasm. The gastrointestinal tract (35 to 65%) and the genitourinary tract (12 to 35%) were the most common sites of primary neoplasia [6]. The main location may not be detected in roughly 30% of patients [6].

The ovaries (14 to 41%), endometrial (1 to 10%), and pancreatobiliary tree (12%) were the most commonly described main sites in women in the literature [7].

SMJN's mechanism is unknown, but some theories have been offered, such as a direct expansion of the tumor, lymphatic dissemination, or hematogenous spread [8].

An SMJN can take many forms, ranging from a soft, harmless nodule to an uneven, painful mass [9].

An umbilical mass, in fact, can be the earliest indicator of an intra-abdominal or pelvic tumor with peritoneal metastases, and it is easy to miss during a physical exam. As a result, early detection and appropriate treatment are critical.

In the presence of an umbilical mass, other diagnoses such as umbilicitis, umbilical abcess, umbilical hernia, umbilical endometriosis, omphalith, keloid, pyoderma gangrenosum, or foreign body can be made.

To confirm the diagnosis of SMJN, a biopsy and histological testing are required. Adenocarcinoma is the most common histological type in about 75% of SMJN cases with intra-abdominal malignancy [2].

An ovarian cancer with intraperitoneal metastases, one of which extends via a hernial supraumbilical orifice, was the final pathological diagnosis in our case.

Although the therapeutic strategy has not yet been standardized, it must be multidisciplinary. Treatment for ovarian cancer is mainly palliative care; however, occasional cases of aggressive treatment with surgery and chemotherapy have been recorded with higher long-term survival [10].

The primary surgical treatment in our case was the removal of the umbilical tumor with the reintroduction of the omentum into the abdominal cavity. It is possible that adjuvant chemotherapy will be required.

Umbilical metastasis is usually associated with a dismal prognosis, with just 15% of patients surviving for two years [10, 11].

## 4. Conclusion

An SMJN is a rare yet significant illness that is frequently misdiagnosed.

Early detection and diagnosis of the original lesion can lead to more effective treatment and a higher rate of survival.
